# Diversity in the Cow Ultralong CDR H3 Antibody Repertoire

**DOI:** 10.3389/fimmu.2018.01262

**Published:** 2018-06-04

**Authors:** Jeremy K. Haakenson, Ruiqi Huang, Vaughn V. Smider

**Affiliations:** Department of Molecular Medicine, The Scripps Research Institute, La Jolla, CA, United States

**Keywords:** antibody, ultralong CDRH3, cow immunoglobulin, activation-induced cytidine deaminase, antibody repertoire

## Abstract

Typical antibodies found in humans and mice usually have short CDR H3s and generally flat binding surfaces. However, cows possess a subset of antibodies with ultralong CDR H3s that can range up to 70 amino acids and form a unique “stalk and knob” structure, with the knob protruding far out of the antibody surface, where it has the potential to bind antigens with concave epitopes. Activation-induced cytidine deaminase (AID) has a proven role in diversifying antibody repertoires in humoral immunity, and it has been found to induce somatic hypermutation in bovine immunoglobulin genes both before and after contact with antigen. Due to limited use of variable and diversity genes in the V(D)J recombination events that produce ultralong CDR H3 antibodies in cows, the diversity in the bovine ultralong antibody repertoire has been proposed to rely on AID-induced mutations targeted to the IGHD8-2 gene that encodes the entire knob region. In this review, we discuss the genetics, structures, and diversity of bovine ultralong antibodies, as well as the role of AID in creating a diverse antibody repertoire.

## Antibody Overview

Antibodies, also known as immunoglobulins (Ig), are an essential and defining characteristic of the vertebrate immune system. They act by binding and inhibiting foreign substances in the body, such as viruses and bacteria, and then destroying them through complement-dependent cytotoxicity, antibody-dependent cell-mediated cytotoxicity, or antibody-dependent cellular phagocytosis (1, 2). A typical antibody is composed of two heavy chains (HC) and two light chains (LC) bound together through both non-covalent interactions and disulfide bonds. There are five classes of HC, each with a distinct function: IgM, IgD, IgG, IgA, and IgE. In most vertebrates, these HCs can pair with two different classes of LCs: lambda (λ) and kappa (κ). Both HCs and LCs are composed of variable and constant regions. The variable region binds antigen and the constant region dictates which downstream effects will occur, such as activation of complement or the recruitment of macrophages, neutrophils, mast cells, basophils, cytotoxic T cells, or natural killer cells. HCs typically have one variable region and three or four constant regions, while LCs have one variable region and one constant region.

**Figure 1:**
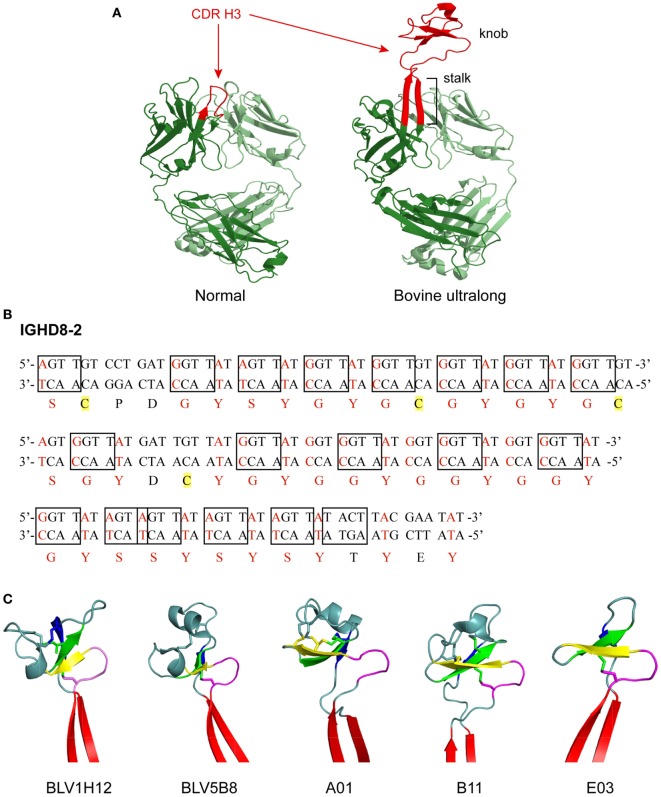
Structure and genetics of ultralong complementarity determining region (CDR) H3 cow antibodies. **(A)** Comparison of normal and ultralong CDR H3 antibody fab fragments. Crystal structures of Yvo, a typical human antibody (left), and B11, an ultralong bovine antibody (right). The CDR H3 of each antibody is highlighted in red. Heavy chains are colored dark green and light chains are colored pale green. Note the two long β strands that make up the stalk of the bovine ultralong CDR H3 and the disulfide-bonded knob domain at the top. PDB IDs of these two fabs are Yvo: 2AGJ; B11: 5IHU. **(B)** Sequence and potential diversity of the ultralong D_H_ region. Potential AID-induced somatic hypermutation hotspot motifs (“RGYW”/“WRCY”) in bovine germline IGHD8-2 are boxed on both strands of the DNA sequence. Nucleotides within a codon that can be mutated to a cysteine-encoding codon with just a single nucleotide change are colored red, and corresponding amino acids encoded by these codons are also colored red. Four cysteine residues encoded by the germline IGHD8-2 are highlighted yellow. **(C)** “Stalk and knob” structures of the ultralong CDR H3s. The β-ribbon stalk is colored red, the type I β-turn is colored magenta, and the three antiparallel β strands are colored yellow, green and blue, respectively. Disulfide bonds formed within the knob region are represented as sticks. PDB IDs of these five fabs are BLV1H12: 4K3D; BLV5B8: 4K3E; A01: 5ILT; B11: 5IHU; and 5IJV.

The segments of the variable region that bind antigen are termed complementarity determining regions (CDRs), and each HC and LC contains three CDRs. These CDRs usually form loops, and together, the six CDRs (three from the HC and three from the LC) form the antigen binding site of an antibody. The third CDR of the HC (CDR H3) often forms the most significant contact with antigen, is longer than the other CDRs, and usually plays a prominent role in antigen binding. In most species, the CDR H3 forms a simple loop structure, which is typically 8–16 amino acids long in humans. In a significant departure from antibodies of most vertebrates, about 10% of the antibodies found in cows have an ultralong CDR H3 ranging from 40 to 70 amino acids in length (3–9) (Figure 1A). For these unusual ultralong CDR H3 bovine antibodies, it is likely that only CDR H3 binds antigen, while the other CDRs play a merely structural role (10, 11).

Some vertebrate species other than cows also contain unique antibody structures. For example, camels and llamas contain antibodies composed of HC homodimers that do not have an LC (12, 13). Interestingly, the CDR H3s of these antibodies often contain a free cysteine which can form a disulfide bond with a cysteine in CDR H1 (12). Sharks also generate antibodies composed of HC homodimers with no LCs called immunoglobulin new antigen receptors, which consist of one variable domain and five constant domains (14–16).

The variable region of an antibody is encoded by variable (V), diversity (D), and junction (J) gene segments (V_H_, D_H_, and J_H_ for the HC genes, V_L_ or J_L_ for the light chain genes which can be specifically denoted V_λ_, V_κ_, or J_λ_, and J_κ_, respectively). In humans and mice, antibody repertoire diversity is achieved through V(D)J recombination, which occurs prior to antigen exposure, and somatic hypermutation (SHM), which occurs after antigen exposure. In V(D)J recombination, typically any V_H_ to D_H_, or D_H_ to J_H_ recombination event can occur to form the full length gene encoding a HC. Since LCs do not contain D genes, any V_L_ gene can combine with any J_L_ gene to form the LC variable region gene. D–J recombination occurs first at the HC locus, followed by V–DJ joining. Further diversity is created during V(D)J recombination through the addition and/or deletion of nucleotides at the junctions between V and D, D and J, or V and J gene segments. Two different types of nucleotide additions can contribute to this junctional diversity: palindromic P nucleotides can be added first, followed by random N nucleotide insertions by terminal deoxynucleotidyl transferase (TdT) (17). Once an organism has been exposed to antigen, SHM further contributes to antibody diversity during the affinity maturation process. This will be described in depth in later sections of this review as it appears critically important to diversity generation in the cow. The sequential nature of V(D)J recombination to form the primary repertoire followed by SHM after exposure to antigen is treated as dogma in molecular immunology, likely due to the murine and human model systems that predominate in immunology research. However, it is clear that some vertebrates, including cows, utilize SHM to diversify the primary repertoire (18–20).

Antibody gene transformations occur in parallel with development of the B cells that produce them. In the bone marrow, hematopoietic stem cells differentiate into pro-B cells, in which V(D)J recombination occurs. These pro-B cells further differentiate into pre-B cells, which make rearranged HCs paired with a LC proxy termed the surrogate light chain. Pre-B cells further differentiate into immature naïve B cells, which produce cell surface IgM paired with a conventional LC. These immature naïve B cells then differentiate into mature naïve B cells that co-express membrane IgM and IgD and can be found throughout the body, especially in the lymphatic system and spleen. Finally, after exposure to antigen, often with the help of T cells, mature naïve B cells proliferate and differentiate into antibody-secreting effector plasma cells or into memory B cells, which produce affinity-matured antibodies of all of the Ig isotypes. It is unclear whether the unusual cow antibodies described below have equivalent distributions in all isotypes and tissue compartments. This is an area of active research.

## The Bovine Antibody Repertoire

The germline genetic components of the bovine antibody repertoire are limited compared to humans, mice, and other vertebrates. At the HC locus, cows have 12 functional V_H_, 23 D_H_, and 4 J_H_ gene segments, while humans have 36–49 V_H_, 23 D_H_, and 6 J_H_ gene segments (10). This means that, in theory, there are up to 6,762 possible VDJ combinations in humans compared to only 920 in cows, allowing for much less combinatorial diversity in the cow.

At the LC lambda locus, cows have 25 V_λ_ and 3 J_λ_ gene segments compared to 73–74 V_λ_ and 7–11 J_λ_ gene segments in humans. The bovine κ locus contains 8 V_κ_ and 3 J_κ_ gene segments compared to 31–36 V_κ_ and 5 J_κ_ gene segments in humans (21). It has been observed that cows preferentially use λ LCs over κ (22), perhaps due to the increased potential for diversity (21).

Bovine antibodies with ultralong CDR H3 occupy at least 10% of the entire bovine antibody repertoire (5, 9), and they are found in all bovine Ig isotypes (23). However, the length distribution of ultralong CDR H3 in bovine Ig isotypes is different (9). It has been established that bovine ultralong antibodies almost always use the same HC V gene segment (IGHV1-7) and the same HC D gene segment (IGHD8-2), which is longer than any other D gene segment known to exist in nature (6, 8–10, 24). In addition, ultralong HCs appear to preferentially pair with λ LCs containing the V30 gene segment which contains residues that bind and may stabilize the ultralong CDR H3 of the HC (9, 25). This restricted use of V and D gene segments suggests that the diversity seen in bovine ultralong antibodies may not primarily result from V(D)J recombination.

## Genetics of Bovine Ultralong CDR H3 Antibodies

Distinct features of germline IGHV1-7 and IGHD8-2 genes facilitate the formation of ultralong CDR H3 in cow antibodies. Nucleotide alignment of IGHV1-7 with other functional IGHV genes reveals a eight-nucleotide duplication, “TACTACTG” at its 3′ end (10). This duplication changes the reading frame at its 3′ end, and results in a longer V-region (encoding “TTVHQ” instead of “AR/K”), which contributes to the initiation of the ascending β-strand of the CDR H3 “stalk” (see the next section). Moreover, compared to other IGHV genes used in cow, IGHV1-7 features low amino acid variability in mature CDR H1 and H2 sequences, which are usually involved in antigen binding in antibodies with short CDR H3 in other species (10). Therefore, the diversity of the cow ultralong antibody repertoire is heavily concentrated in the CDR H3 which is mainly encoded by the IGHD8-2 gene.

IGHD8-2 encodes the majority of the ultralong CDR H3 after the “TTVHQ” motif. It encodes 48 amino acid residues and features 19 activation-induced cytidine deaminase (AID) induced SHM hotspots (“RGYW or WRCY” discussed later) (9, 10) (Figure 1B). In addition, IGHD8-2 contains 4 cysteine codons and another 38 codons that can readily be mutated to cysteine with just one nucleotide change (9) (Figure 1B). These existing and potential cysteine codons would enable disulfide bond formation within the CDR H3, and thus diversify the bovine ultralong antibodies at the structural level (9).

## Structural Features of Bovine Ultralong CDR H3 Antibodies

Crystal structures of five bovine antibody Fab fragments with ultralong CDR H3 regions have been published (9, 25). Despite significant amino acid differences, as well as different numbers of amino acids in the CDR H3 region of these five antibodies, all of them adopt the same general CDR H3 structure, where a β-ribbon stalk supports a knob region that contains one type I β-turn, one conserved disulfide bond, and three antiparallel β strands (Figure 1C). The ascending β-strand in the stalk is initiated by the extended C-terminus encoded by IGHV1-7, and the descending β-strand is contributed by the C-terminus encoded by IGHD8-2, while the knob region is encoded solely by IGHD8-2. The supported knob region extends far out from the traditional antigen binding surface generated by the other five CDRs and has the potential to bind antigens with concave epitopes (9, 11, 25).

Although these ultralong CDR H3 antibodies share the general “stalk & knob” scaffold, each antibody also possesses distinct structural variations in the CDR H3 (Figure 1C). These structural variations are reflected in differences in the length of the stalk and the orientation of the knob relative to the rest of the antibody structure (25). For example, the stalk length of BLV1H12 is the longest among these five antibodies, while the stalk length of A1 is the shortest (25). When the five Fab structures are superimposed by the shared type I β-turn and the three antiparallel β strands in the knob region, obvious differences in stalk positions are observed, reflecting different knob orientations that are supported by the stalks (25). Furthermore, as the number and positions of cysteine residues in the knob region differ significantly among these cow ultralong antibodies, each of them possesses a knob region with unique disulfide bond patterns (25). Therefore, different stalk lengths, knob orientations, and disulfide bond patterns, in addition to diverse amino acid content within the knob regions of these antibodies provide remarkable structural diversity generated in the bovine ultralong CDR H3 antibody repertoire.

## Diversity and Development of the Bovine Ultralong CDR H3 Antibody Repertoire

**Figure 2:**
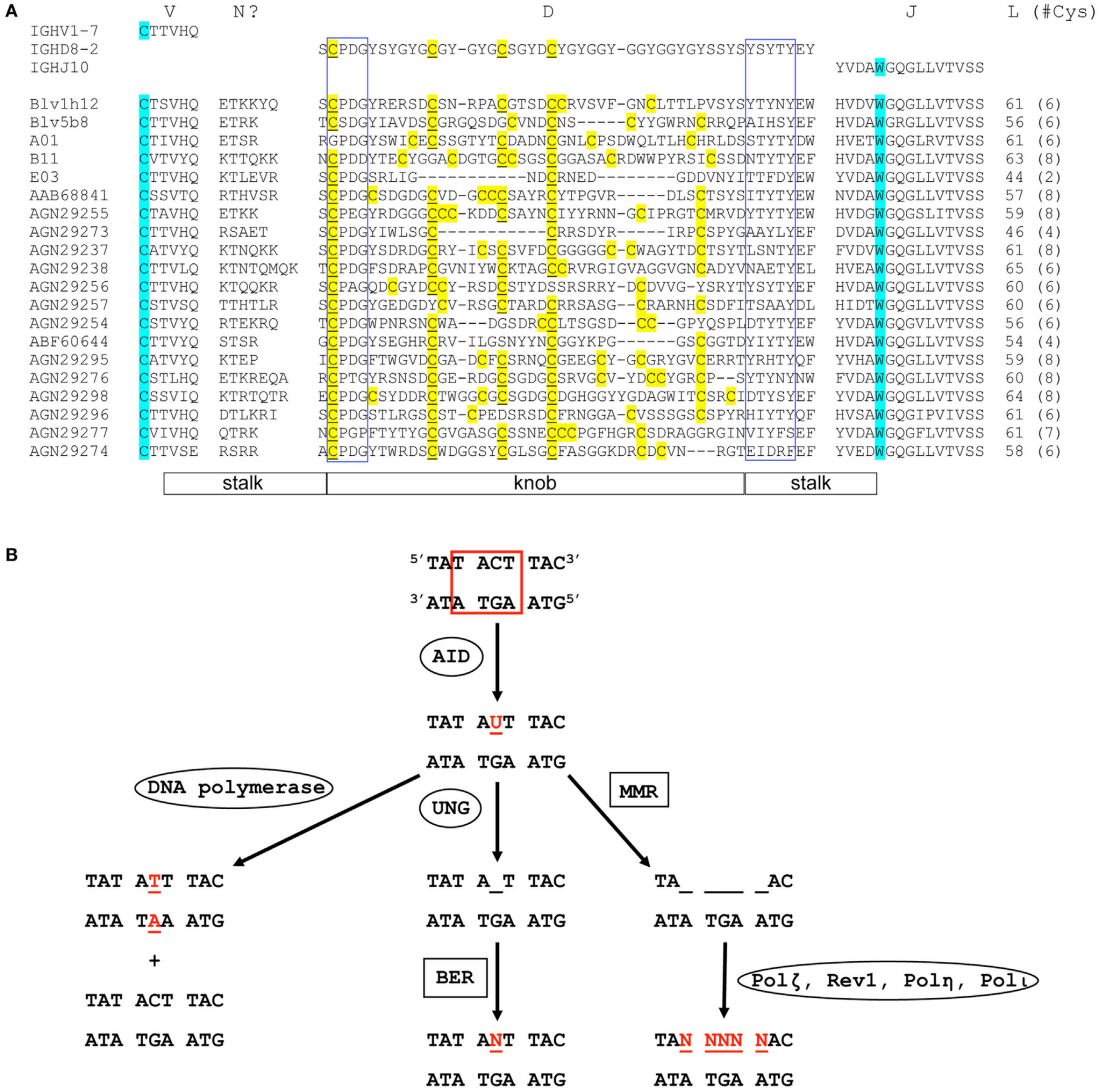
Sequence diversity and genetic mutation in ultralong complementarity determining region (CDR) H3 regions. **(A)** Alignment showing diversity of bovine ultralong CDR H3. Residues encoded by germline IGHV1-7, IGHD8-2, and IGHJ10 are shown on the top of the alignment for reference. The start and the end of the CDR H3 are defined by the conserved cysteine and tryptophan which are colored turquoise. *N*? indicates residues possibly encoded by terminal deoxynucleotidyl transferase catalyzed *N* additions or conserved short nucleotide sequence insertions at the V–D junction. Cysteine residues in the D region are colored yellow and the unmutated germline-encoded cysteines are also underlined. The conserved CPDG and YxYxY motifs near the beginning and the end of the D region are enclosed in blue boxes, respectively. The length of each CDR H3 and the number of cysteine residues (in parenthesis) it contains is shown on the right of the alignment. The stalk and knob regions are indicated below the alignment. The alignment was performed using MAFFT, then manually adjusted to align the conserved residues. The first five sequences with crystal structures available are named according to published nomenclature, and the remaining sequences are named with their GenBank accession numbers. **(B)** Mutation induction by AID. AID deaminates cytosine to uracil at an AID hotspot (boxed in red). This can be followed by three different processes that cause point mutations. DNA polymerase replicates in both strands of parental DNA, resulting in two daughter strands, one with a C to T mutation and one without (Left). Uracil-DNA glycosylase removes uracil, creating an abasic site (Middle). Error-prone base excision repair can then replace this site with any dNTP (*N* = A, C, G, or T). Mismatch repair removes several nucleotides around and including the uracil (right). This is then followed by error-prone DNA polymerases, which can cause a mutation at any of the sites, where nucleotides have been removed (Ovals designate single enzymes and squares designate multi-enzyme processes). Mutated nucleotides are shown in red.

Although combinatorial antibody diversity generated through V(D)J recombination in the cow is limited, some diversity of these ultralong CDR H3 antibodies can arise from junctional diversity; P-nucleotide insertions, and TdT-catalyzed N additions and deletions can potentially occur at the V–D and D–J junctions (8, 26). It has been reported that some ultralong CDR H3 antibody genes can gain additional diversity through conserved short nucleotide sequence insertions (up to 6 codons) at the V–D junction (8). Importantly, the diversity of the ultralong CDR H3 antibody repertoire may be primarily generated through extensive SHM mediated by AID, which can occur either before or after antigen contact (Figure 2). For example, AID is expressed in naïve B cells in fetal bovine ileal Peyer’s patches (lymphoid follicles in the small intestine) and spleen, where it induces SHM by mutating the CDR regions more often than the framework (FR) regions, a pattern similar to AID-induced SHM upon antigen contact in adult B cells (20). It has also been proposed that AID may mediate a novel diversification mechanism in cow either by deleting interior codons in the IGHD8-2 region without disrupting the knob and stalk structure, or by varying the number of cysteine residues in the knob, thus creating different disulfide bond patterns (Figure 2A) (9, 10, 25). Given that relatively limited diversity is generated by recombinational mechanisms in the bovine ultralong CDR H3 antibody repertoire, AID may be the primary driver for diversity development in the bovine ultralong CDR H3 antibody system.

## Evolutionary Origin of AID

AID, which drives antibody class switch recombination (CSR) (27), SHM (27), and gene conversion (GC) (28), plays an important role in vertebrate immune responses and antibody diversification, and may play a particularly important role in diversifying cattle antibody genes. Given the potential unique importance of AID in the cow antibody repertoire, here we will review AID, whose activity and characteristics have been mostly studied in mouse and human systems, but whose properties are likely also applicable to bovines. AID belongs to the AID/APOBEC family which includes AID and APOBEC1 to APOBEC4. Although *in vivo* functions of APOBEC2 and APOBEC4 are still unclear, the other three members are zinc-dependent cytidine deaminases that catalyze the deamination of cytidine (C) to uracil (U) in either DNA (AID and APOBEC3) and/or RNA substrates (APOBEC1) (29). The first member characterized in this family was APOBEC1, which is an RNA editing enzyme that deaminates C6666 to U in the apolipoprotein B pre-mRNA in the small intestine, resulting in an expressed truncated apolipoprotein B, in contrast to the longer form which is synthesized in the liver (30, 31). AID, as a close relative of APOBEC1, is a 24 kDa enzyme that shares a similar structural and catalytic scaffold to APOBEC1, but only catalyzes deamination of deoxycytidine (dC) to deoxyuracil (dU) in either single-stranded DNA *in vitro* (32) or double-stranded DNA during transcription *in vivo* (33). APOBEC3, another DNA deaminase, fights intruding retroviruses by deaminating dC to dU on the reverse transcribed minus strand of viral DNA (34–37).

AID is encoded by the *AICDA* gene in humans, mice, and cows (38). The existence of AID is likely ancient because *AICDA* homologs have been identified in jawless vertebrates, in which immunoglobulin-type antigen receptors have not evolved (39–42). Indeed, lampreys use variable lymphocyte receptors, which contain leucine-rich repeat (VLR) modules that are structurally distinct from immunoglobulins, as the antigen receptors to carry out adaptive immune responses (39, 40). The diversity of the VLR repertoire comes from somatic insertions and rearrangements of different LRR modules from a large LRR module pool into the germline VLR gene, and AID was proposed to contribute to this GC-like process (39, 40, 42–44). The ability of AID to induce SHM evolved early in lower vertebrates (e.g., cartilaginous and bony fish) (45). Furthermore, although AID-induced CSR evolved later in tetrapods, fish AID was pre-adapted to drive CSR as ectopic expression of zebrafish AID in AID knockout mice is able to mediate equivalent CSR and SHM in murine immunoglobulin genes compared to murine AID (46, 47). In contrast, APOBEC1 and 3 evolved recently only in mammals (29, 48). APOBEC2 and 4 are likely ancient members in the family as well because they also exist in jawless vertebrates (but not in lamprey), similar to AID (48). In addition, phylogenetic relationships, gene structures, and chromosome loci of AID/APOBEC family members suggest that APOBEC1 and 3 likely arose from gene duplications of AID in mammalian species, and RNA editing deaminase activity likely evolved from ancestral DNA deaminase activity (48). Therefore, the activities of AID on immune system genes may have been the most ancient activity of this family of enzymes, and is important in apparently being the most ancient diversification enzyme for immune receptors.

## Tissue and Cellular Expression of AID

AID was first discovered in CH12F3-2 murine B lymphoma cells (49). Much early work on AID also used the DT40 chicken bursa lymphoma cell line (50). Some of the first experiments on AID found that it is expressed in Peyer’s patches and lymph nodes, and to a lesser extent in the spleen and bone marrow (49). Microscopically, AID is expressed in germinal centers, which are regions where B cells proliferate and differentiate in response to antigen (49). This expression pattern is consistent with AID’s role in affinity maturation, GC, and CSR, processes that take place in B cells in the lymphatic system and spleen.

## Function and Activity of AID

AID is a remarkable enzyme because of the many activities that it is responsible for, which include SHM, CSR, and GC. In SHM, AID introduces point mutations into the variable region of an antibody gene by deaminating cytosine to uracil. Several processes can take place following this C to U mutation (Figure 2B). In one case, DNA replication can replace the uracil with a thymidine, resulting in two different daughter strands: one with a T-A base pair, and one with the original G-C base pair. In another case, uracil-DNA glycosylase (UNG) can remove the U, creating an abasic site, which can then be replaced by any nucleotide (A, G, C, or T) through the process of short patch base excision repair (BER). These two processes combined account for about 40% of AID-mediated mutations (51). A third possibility is that mismatch repair (MMR) can excise several nucleotides (nt) around and including the U. When this is followed by an error-prone polymerase, such as Polζ, Rev1, Polη, or Polι (52, 53), mutations can occur at any of the excised positions on the DNA (54). This third process accounts for about 60% of AID-mediated mutations (51). Thus, AID catalyzes a C to U mutation that can ultimately lead to additional mutations near the original deaminated cytosine. These point mutations can in turn alter the amino acid sequence of the antibody, changing its affinity for its antigen. The CDR H3 region of mature cow ultralong CDR H3 antibodies are enormously mutated, thus any or all of these activities of AID may play a role in shaping the repertoire.

The mutation rate of AID is in the range of 0.78–10.4 mutations per 10^4^ base pairs (55, 56). Importantly, SHM is up to six times as likely to occur in SHM hotspot regions compared to other regions of DNA (57). Canonical SHM hotspots have the sequence motif RGYW (R = purine, Y = pyrimidine, W = A or T) or its reverse complement WRCY (56), although DGYW (D = A, G, or T)/WRCH (H = A, C, or T) may be a better predictor of SHM (58). The significant concentration of SHM hotspots in the bovine IGHD8-2 gene segment suggest that AID may preferentially act on this gene segment, thereby creating diversity in the bovine ultralong antibody repertoire. Indeed, IGHD8-2 contains 19 AID hotspots, including 38 out of 48 (79%) amino acid codons that can mutate to cysteine with a single nucleotide mutation. Thirty of those codons are at least partially within AID hotspots (9) (Figure 1B), suggesting that AID plays an important role in creating cysteine diversity in the ultralong D region.

In addition to the introduction of point mutations, insertions and deletions also occur during SHM. These insertions and deletions typically occur in CDRs and are twice as likely to occur at SHM hotspots compared to other regions of DNA (59), implicating AID in this process. Insertions typically duplicate the preceding codons (e.g., AGC to AGC AGC) (60) and usually range from 2 to 23 nt in length (60, 61), while deletions are usually 1 to 51 nt long (61). Frameshift insertions and deletions (i.e., those not divisible by 3) can result in premature stop codons, so that often only in-frame insertions and deletions survive clonal selection. Ultralong CDR H3 bovine antibodies often contain deletions ranging from 3 to 54 nt in the D region (10). Deletions in bovine ultralong antibodies are easier to detect than in short antibodies because in short antibodies, it is difficult to ascertain whether an insertion/deletion has occurred or whether a different V, D, or J gene segment has been used, particularly when the CDR H3 has been highly mutated. Bovine ultralong antibodies, on the other hand, appear to always use the IGHD8-2 germline gene segment, which are 48 amino acids long. By comparison, the next longest bovine DH gene segment (IGHD7-4) is only 23 amino acids long. The rate of deletions in bovine ultralong antibodies has been reported to be 48% (10), compared to 2.6% in human IgG antibodies (62). However, this latter number might be an underestimate due to difficulty inherent in detecting insertions and deletions in short CDR H3 antibodies. Clearly, the ability of AID-mediated insertions and deletions to increase antibody variability further highlights the importance of AID and SHM in creating diversity in bovine ultralong antibodies.

In addition to causing SHM, AID is also responsible for CSR, in which the constant region of an antibody switches from one class to another (e.g., IgM to IgG) while the variable region remains the same. Indeed, mice and humans deficient in AID exhibit hyper-IgM syndrome, in which the immune system is unable to make IgG, IgA, and IgE antibodies (27, 63). CSR takes place during transcription of antibody genes. While antibodies are being transcribed, R-loops form, in which one strand of DNA is hybridized to the resulting mRNA and the other strand of DNA is in a single-stranded state (ssDNA). During transcription, AID targets R-loops and deaminates C to U on both strands of DNA. UNG then removes the U, creating an abasic site that is converted into a single-strand nick by apurinic/apyrimidinic endonuclease. In effect, nicks on both strands of DNA create a double-strand break (64). When double-strand breaks occur in the switch regions that flank each constant region, the existing constant region is removed and replaced by a new constant region through a DNA synaptic process involving non-homologous end joining (NHEJ) (64–66). Of note, hotspots occur in close proximity (e.g., separated by only two base pairs) or even overlap in the cow IGHD8-2 (Figure 1B), suggesting the possibility that double-strand breaks, and associated deletions/insertions could occur through NHEJ within the ultralong D region.

Besides catalyzing SHM and CSR, AID also mediates GC, a process found in chickens and rabbits but not apparently in humans and mice (50). In GC, which takes place after V(D)J recombination but before SHM, pseudogenes on the same chromosome as the antibody genes are used as a template for part of the antibody variable region during DNA replication, thus adding diversity to the antibody (50). Bovine HCs and LCs have been reported to undergo GC (67, 68). However, some researchers suggest that AID-mediated SHM alone accounts for bovine Ig diversification after V(D)J recombination (69). As there are several V_H_ pseudogenes in the cow, it will be important to definitively determine whether GC plays a role in diversity generation in bovines.

## Regulation of AID

AID is primarily located in the cytoplasm (70) but clearly functions in the nucleus of B cells (71). Similar to its close relative, APOBEC1, AID is also a nuclear–cytoplasm shuttle protein that contains a nuclear localization signal on its N-terminus and a nuclear export signal on its C-terminus. Both signals are indispensable for maintaining the dynamic equilibrium of AID distribution between the nucleus and the cytoplasm (71, 72). As a potential oncogenic mutator which can also target non-Ig genes in cells other than B cells, the cellular level of AID has to be tightly controlled to prevent diseases caused by its abnormal expression (72, 73). AID expression is induced through various B cell signaling pathways, including B cell receptor, IL-4R, toll-like receptor, and CD40 (38). Several levels of regulation then engage to control the AID expression process. First, AID has very long transcriptional regulatory elements including a 1.6 kb promoter with which transcription factors NF-κB, HOXC4, and Pax5, etc., interact, thus regulating its transcription (74–76). Second, after transcription, cellular AID mRNA levels are modulated by two microRNAs (miR-155 and miR181b) (77–79). Third, the amount of AID is also regulated at the post-translational level in both the cytoplasm and the nucleus. For example, Hsp90 is able to stabilize AID in the cytoplasm of B cells by binding to and preventing it from being degraded through polyubiquitination (80). Translation elongation factor 1α also forms a complex with AID in the cytoplasm to prevent excessive transport into the nucleus (81), while REG-γ mediates degradation of overexpressed AID in the nucleus through proteasomes (82). Moreover, activities of AID are further regulated through phosphorylation. For example, protein kinase A phosphorylates AID at Ser-38, which is crucial for the ability of AID to induce CSR (83, 84). Given that bovines induce SHM in the primary repertoire, these regulatory mechanisms are likely important in cow B-cell development, however, detailed study of AID regulation, and potential differences between human, mouse, and cow, remain to be elucidated.

The molecular mechanism of how AID is specifically targeted to the Ig variable regions to induce SHM is still under exploration. The previously described SHM hotspot motif (“RGYW”) alone is not enough to explain why Ig loci are preferentially targeted by AID, because this motif also exists in many other functional non-Ig genes (85). One early study investigating AID targeting on different segments of Ig genes concluded that the reason for the first 100 bp at the 5′ end and C regions of the Ig genes free of SHM is due to spatial constraints which prevent AID’s access to these regions (85, 86). Another proposed positive regulation for AID targeting to the Ig variable regions is the E-box motif (“CANNTG”), a cis-regulatory element (CRE) that exists at multiple locations in the Ig loci (87). E-box motifs provide binding sites for the E2A trans-acting factors, which stimulate SHM during transcription (79, 85, 87, 88). However, it has been reported that E2A proteins may also interact with other unknown cis-acting elements, not the E-box motif alone (88, 89). To date, the only confirmed SHM-related CRE is the one that targets AID to the Ig light chain locus in chicken DT40 cells to induce SHM. It is located in the 3′ regulatory region and is different from previously known CREs (79, 90–92). However, how AID interacts with this CRE and whether similar CREs exist in chicken HC locus as well as in other mammalian B cells such as cows still await further characterization. One latest research effort made use of a DNA capture library to identify 275 AID targets at the genomic level in mouse germinal center B cells and successfully revealed more characteristics of AID targets (93). For example, AID targets have higher transcription levels and rates than non-targets, higher binding density of RNAPolII and Spt5, and more transcription and elongation marks. AID targets are also close to superenhancers as well as within convergent transcription regions (93). In addition, this research also confirms the roles of BER and MMR in repairing AID-mistargeted genes in germinal center B cells (93, 94). The findings and methods in this research contribute substantially to the understanding of AID targeting specificity, and is likely applicable to bovine germinal centers.

## Potential Target of AID in Bovine Ultralong CDR H3 IG Genes

Because bovine antibodies with ultralong CDR H3 almost solely use IGHV1-7, which encodes antibodies with low variation in CDR H1 and H2, AID has been hypothesized to preferentially target IGHD8-2, which encodes the entire knob region, to induce SHM (9, 10). This targeting occurs in the pre-immune repertoire (before antigen exposure). As discussed in previous sections, evidence supporting this hypothesis includes the prevalence of significant hotspot motifs (“RGYW”) in the germline IGHD8-2 sequence (9), diverse amino acid content, length variation, and different cysteine numbers and positions in the knob regions encoded by IGHD8-2 (9, 10). Experiments on AID’s ability to induce mutations, insertions, and/or deletions into the bovine germline IGHD8-2 gene will be important to ascertain its role in development of the cow ultralong CDR H3 repertoire.

## Summary

A subset of bovine antibodies contains ultralong CDR H3s ranging from 40–70 amino acids in length. These ultralong CDR H3s form a unique stalk and knob structure that likely allows them to bind epitopes that would be inaccessible to shorter antibodies. Ultralong CDR H3s always contain the same V and D gene segments, implying that a significant component of their diversity must result from processes other than V(D)J recombination. Indeed, the germline D gene segment found in all ultralong antibodies contains 19 AID hotspots, suggesting that AID is largely responsible for the unusual cysteine-based diversity seen in bovine ultralong antibodies, possibly through a combination of point mutations, insertions, deletions, and GC.

## Nomenclature

**Table:** 

ADCC	Antibody-dependent cell-mediated cytotoxicity
AID	Activation-induced cytidine deaminase
APE1	Apurinic, apyrimidinic endonuclease
BER	Base excision repair
C	Cytidine
CDC	Complement-dependent cytotoxicity
CDR	Complementarity determining region
CSR	Class switch recombination
CRE	cis-regulatory element
D	Diversity
FR	Framework
GC	Gene conversion
HC	Heavy chain
Ig	Immunoglobulin
IgG	Immunoglobulin gamma
IgNAR	Immunoglobulin new antigen receptor
J	Joining region
LC	Light chain
LRR	Leucine-rich repeat
mAb	Monoclonal antibody
MMR	Mismatch repair
NHEJ	Non-homologous end joining
nt	Nucleotide
Polη	DNA polymerase eta
Polι	DNA polymerse iota
Polζ	DNA polymerase zeta
SHM	Somatic hypermutation
TdT	Terminal deoxynucleotidyl transferase
U	Uracil
UNG	Uracil-DNA glycosylase
V	Variable
V_H_	Heavy chain variable region
V_L_	Light chain variable region
VLR	Variable lymphocyte receptor

## Author Contributions

JH, RH, and VS wrote the manuscript and generated the figures.

## Conflict of Interest Statement

The authors declare that the research was conducted in the absence of any commercial or financial relationships that could be construed as a potential conflict of interest.
